# Validation of Guanidine-EDTA as a Preservative Agent for the Analysis of miRNAs and mRNAs in Blood Samples of Chagas Disease Patients

**DOI:** 10.3390/pathogens15040424

**Published:** 2026-04-14

**Authors:** Amanda Faier-Pereira, Paula Finamore-Araujo, Maria Mikaely Ribeiro Brito, Alejandro Marcel Hasslocher-Moreno, Otacilio C. Moreira

**Affiliations:** 1 Platform of Molecular Analysis, Laboratory of Molecular Virology and Parasitology, Oswaldo Cruz Institute, Oswaldo Cruz Foundation, Rio de Janeiro 21040-360, RJ, Brazil; 2 Evandro Chagas National Institute of Infectious Diseases, Oswaldo Cruz Foundation, Rio de Janeiro 21040-360, RJ, Brazil

**Keywords:** Chagas disease, *Trypanosoma cruzi*, molecular diagnosis, biomarker, microRNAs, RNAs, guanidine-EDTA

## Abstract

Chagas disease (CD) is a neglected tropical disease caused by the flagellate protozoan *Trypanosoma cruzi*, representing a major socioeconomic challenge. MicroRNAs (miRNAs) are small non-coding RNA molecules that regulate gene expression, and several pathogens, including *T. cruzi*, can modulate host miRNA networks. In this context, we hypothesized that host-derived miRNAs could serve as biomarkers in chronic CD. Given the intrinsic lability of RNA, we evaluated the efficacy of a 6 M guanidine-HCl/0.2 M EDTA solution, widely used in the molecular detection of *T. cruzi* DNA, in preserving mRNAs and miRNAs when mixed in a 1:1 ratio with human blood. Samples with or without guanidine were enriched with exogenous miRNAs (cel-miR-39 and cel-miR-54) and stored at 4 °C. RNase P expression was also evaluated in blood samples stored for up to 120 days and in samples from patients with CD, allowing direct comparison of mRNA stability over time. Samples preserved with guanidine-EDTA showed Ct values that were 4 to 5 cycles lower for all targets analyzed and demonstrated greater RNA stability over time. Taken together, these findings demonstrate that guanidine-EDTA robustly preserves mRNA and miRNAs in human blood, expanding the feasibility of molecular analyses in retrospective samples and corroborating its potential application in the studies of biomarkers of therapeutic response and prognosis in CD.

## 1. Introduction

Chagas disease (CD), also known as American trypanosomiasis, is classified among the group of neglected tropical diseases (NTDs) recognized by the World Health Organization (WHO). Its etiology is associated with the flagellated protozoan *Trypanosoma cruzi*, the causative agent of an infection with significant morbidity. It is estimated that approximately 7 million individuals worldwide are infected with this pathogen, mainly in Latin America, with nearly 70 million living in areas at risk of exposure to infection [1].

The cardiac manifestation of CD stands out as one of the leading causes of mortality associated with cardiovascular complications in endemic regions [2]. Currently, no prophylactic or therapeutic vaccine is available. The existing etiologic chemotherapy, based on benznidazole (BZN) or nifurtimox (NTX), shows greater efficacy during the acute phase of infection, with cure rates ranging from 60% to 80%. Regarding the chronic phase, official guidelines establish criteria for trypanocidal treatment based on its potential to slow disease progression and reduce the risk of congenital transmission [3].

The gold standard for assessing the efficacy of CD treatment is seroconversion in conventional serological tests, which may take decades to occur [4,5]. In this regard, the Polymerase Chain Reaction (PCR) has emerged as the primary tool for monitoring patients undergoing treatment. Within this context, PCR must provide rapid, accurate, and accessible diagnoses, making it essential that blood samples are properly stored in a 6 M guanidine-HCl/0.2 M EDTA pH 8.0 solution. This solution is widely used to preserve the integrity of genetic material, enabling more sensitive and specific detection of *T. cruzi* DNA, as demonstrated in molecular diagnostic studies, and allowing DNA extraction and analysis even after long periods of storage [6,7,8,9].

However, the assessment of parasitological cure by PCR faces significant challenges, particularly due to the low and transient parasitemia observed in chronically infected individuals [9,10]. This scenario imposes major limitations on the validation of new drugs for CD, since monitoring parasite load (parasite DNA) is insufficient to ensure cure control. Conversely, the intrinsic lability of RNA has made these molecules particularly suitable as markers of cellular viability, both in host–pathogen interaction systems and in tumor cell contexts [11,12]. Nevertheless, the detection of *T. cruzi* RNA in blood samples from CD patients still requires further elucidation as a potential tool for monitoring parasitological cure in individuals undergoing antiparasitic treatment.

In addition, research indicates that during infection, changes in host cell miRNA expression profiles may signal defense mechanisms or subversion strategies adopted by the pathogen [13]. Several pathogens, including viruses [14,15], bacteria [16,17], Apicomplexan parasites [18,19], *Leishmania* [13], and *T. cruzi* [20,21], have demonstrated the ability to manipulate miRNA networks in infected host cells.

miRNAs are a class of small, single-stranded non-coding RNAs that regulate the expression of target messenger RNAs (mRNAs), either by inhibiting transcription or inducing degradation [22,23]. A single miRNA can target between 250 and 500 different mRNAs, potentially preventing the translation of dozens to hundreds of proteins simultaneously [22].

Thus, the identification of biomarkers to monitor therapeutic efficacy in CD has become a major focus of investigation. Considering the hypothesis that host-derived miRNAs may act as biomarkers of therapeutic response in patients with chronic CD, it becomes evident that it is necessary to evaluate the capacity of the 6 M guanidine-HCl/0.2 M EDTA pH 8.0 solution, widely used in studies related to the molecular diagnosis of CD, to preserve not only DNA but also mRNAs and miRNAs when mixed in equal volume with human blood.

## 2. Materials and Methods

### 2.1. Patient Samples

The study was validated using 10 guanidine-EDTA blood samples from patients with confirmed Chagas disease, based on two positive conventional serological tests, followed at the Instituto Nacional de Infectologia Evandro Chagas (INI/Fiocruz), Rio de Janeiro, Brazil. All participants provided informed consent, and the study was approved by the Ethics Committee (CAAE 0070.0.009.000-07). Samples were previously subjected to boiling according to older protocols for conventional PCR targeting kDNA and have since been stored at 4 °C [8,24]. As negative controls, 10 blood samples from individuals from non-endemic areas, negative for Chagas disease by serology and PCR, were used. These samples were preserved or not in 6 M guanidine hydrochloride and 0.2 M EDTA (1:1) and stored at 4 °C without boiling. No samples were subjected to freeze–thaw cycles, and all were acclimated to room temperature prior to processing.

### 2.2. Total RNA Extraction (miRNAs and mRNAs)

The isolation of total RNA, including microRNAs (miRNAs) and messenger RNAs (mRNAs), was performed from 200 µL of human blood preserved in 6 M guanidine hydrochloride and 0.2 M EDTA solution (GEB), as well as from pure human blood (without guanidine-EDTA). For this procedure, the TRIzol LS reagent (Invitrogen, Thermo Fisher Scientific, Waltham, MA, USA) was used, strictly following the manufacturer’s instructions. Prior to RNA extraction for each sample, 5 µL of two miRNAs were added as exogenous controls: cel-miR-39-3p and cel-miR-54 (synthetic microRNAs derived from the nematode *Caenorhabditis elegans*, used to normalize microRNA expression) at a concentration of 0.5 fmol/µL. At the end of the extraction, RNA was recovered in 30 µL of nuclease-free water. RNAs were quantified using a Nanodrop ND2000 (Thermo Fisher Scientific, Waltham, MA, USA).

### 2.3. DNase Treatment and Control of Genomic DNA Contamination

All extracted total RNA was treated with DNase I, Amplification Grade (Invitrogen, Thermo Fisher Scientific, Waltham, MA, USA) to completely remove potential genomic DNA contaminants before subsequent analyses. To ensure the effectiveness of DNA removal, negative reverse transcription (−RT) controls were systematically included in all experiments. For mRNA analysis by One-Step RT-PCR, control reactions were performed without the addition of the SuperScript III RT/Platinum Taq enzyme. Similarly, for miRNA analysis, reverse transcription reactions aimed at obtaining cDNA were conducted in parallel without the addition of the MultiScribe Reverse Transcriptase enzyme.

### 2.4. Reverse Transcription and microRNA Gene Expression by Quantitative Real-Time PCR

MicroRNA (miRNA) reverse transcription was performed in a total volume of 15 µL in an Eppendorf Mastercycler thermocycler (Eppendorf, Hamburg, Germany) at 16 °C for 30 min, 42 °C for 30 min, and 85 °C for 5 min. Five microliters of miRNA were used, employing the TaqMan MicroRNA Reverse Transcription Kit (Thermo Fisher Scientific) with stem-loop specific primers for cel-miR-39-3p (Assay ID: 478293_mir), cel-miR-54-5p (Assay ID: 462123_mat), and U6 snRNA (Assay ID: 001973).

Real-time RT-qPCR was conducted in a 10 µL reaction containing 5 µL of 2× TaqMan Universal Master Mix II (Applied Biosystems, Thermo Fisher Scientific, Waltham, MA, USA), 0.5 µL of TaqMan Assays (Applied Biosystems) specific for cel-miR-39-3p (Assay ID: 478293_mir), cel-miR-54-5p (Assay ID: 462123_mat), or U6 snRNA (Assay ID: 001973), 2 µL of cDNA, and 2.5 µL of RNase-free water. PCRs were run on a QuantStudio 7 Pro qPCR system (Thermo Fisher Scientific) using the cycling conditions: 10 min at 95 °C, followed by 40 cycles of 15 s at 95 °C and 60 s at 60 °C. Analyses were performed using a threshold set at 0.02 for both miRNAs.

### 2.5. Analysis of mRNA Integrity Using One-Step Reverse Transcription Quantitative PCR (RT-qPCR)

**Figure 1:**
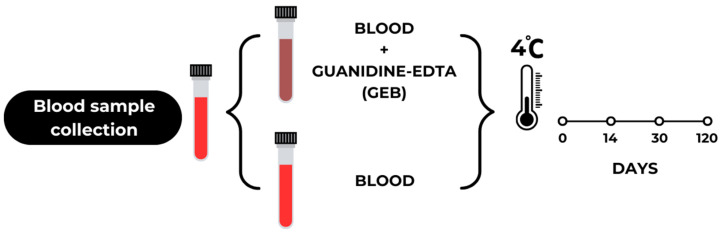
Experimental design for the preparation and storage of whole blood and blood with added guanidine-EDTA (GEB) at 4 °C for 0, 14, 30, and 120 days.

Messenger RNA integrity was investigated using five *T. cruzi*-negative blood samples, in the presence and absence of 6 M guanidine-HCl/0.2 M EDTA, stored at 4 °C for different time periods (0, 14, 30, and 120 days). At each point, total RNA was extracted and subjected to one-step RT-qPCR targeting the human RNase P transcript, used as an endogenous control (Figure 1).

Reactions were performed in technical duplicates in a final volume of 10 µL, containing 2 µL of cDNA, 5 µL of reaction mix [2×], 1 µL of TaqMan RNase P reagent [20×] (Applied Biosystems, cat. no. 4316844), and 0.5 µL of SuperScript III RT/Platinum Taq enzyme (Invitrogen). All assays were carried out on a QuantStudio 7 Pro system (Thermo Fisher Scientific) under the following cycling conditions: 50 °C for 15 min, 95 °C for 10 min, followed by 45 cycles of 95 °C for 15 s and 58 °C for 1 min.

RNA integrity was also assessed by capillary electrophoresis using the TapeStation 4150 system (Agilent Technologies, Santa Clara, CA, USA) using the High Sensitivity RNA ScreenTape Assay (Agilent Technologies, Cat. N°: 5067-5579), following the manufacturer’s instructions. Whole blood samples and guanidine-EDTA-preserved blood samples stored for 120 days at 4 °C were analyzed. The RNA extracted from a mice cardiac tissue sample was included as a positive control and processed in parallel. Electropherograms and RNA Integrity Number (RIN) values were obtained for each sample and used for comparative assessment of RNA integrity across the different experimental conditions.

### 2.6. Statistical Analysis

All samples were analyzed in technical duplicates, from which means, standard deviations, and confidence intervals were calculated. Statistical analyses were performed using SigmaPlot for Windows (SPSS), version 12 (Systat Software, San Jose, CA, USA). To compare miRNA expression levels between two groups, using delta Ct values (mean Ct of the target miRNA—mean Ct of the reference miRNAs), either Student’s *t*-test or the Mann–Whitney Rank Sum test was applied, depending on the parametric or non-parametric distribution of the data, respectively. For the graphic construction and statistical analysis, no amplification was considered Ct = 45 (the total number of the qPCR cycles).

## 3. Results

### 3.1. Analysis of miRNA Integrity in Whole Blood and GEB Samples

Initially, two synthetic microRNAs (cel-miR-39 and cel-miR-54) were evaluated as exogenous internal controls in whole blood and GEB samples. Expression levels were assessed using the comparative Ct method.

**Figure 2:**
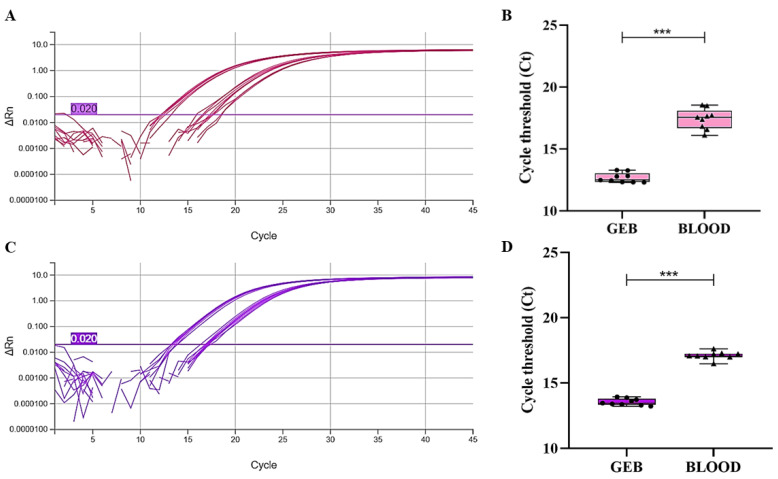
Analysis of exogenous internal control miRNAs in blood samples preserved or not in guanidine-EDTA. (**A**) Amplification curves for the exogenous control cel-miR-39 in GEB and whole blood samples. (**B**) Box plot comparing Ct variations of cel-miR-39 between GEB and whole blood. (**C**) Amplification curves for the exogenous control cel-miR-54 in GEB and whole blood samples. (**D**) Box plot comparing Ct variations of cel-miR-54 between GEB and whole blood. In (**A**,**C**), the purple horizontal line means the qPCR threshold. In (**B**,**D**), the circle and triangle symbols mean the individual Ct value for each sample. *** *p* < 0.001 (one-way ANOVA).

Figure 2 shows the amplification curves of the exogenous internal controls (Figure 2A: cel-miR-39; Figure 2C: cel-miR-54), comparing GEB samples with whole blood samples. Box plots highlight statistically significant differences between the groups for both controls (cel-miR-39 and cel-miR-54; Figure 2B and Figure 2D, respectively). GEB samples exhibited higher analytical sensitivity, reflected by consistently lower Ct values with reduced variability compared to whole blood. Specifically, the mean Ct values observed were 12.675 ± 0.393 for cel-miR-39 and 13.539 ± 0.255 for cel-miR-54 in GEB, whereas in whole blood they were 17.420 ± 0.819 and 17.097 ± 0.304, respectively.

**Figure 3:**
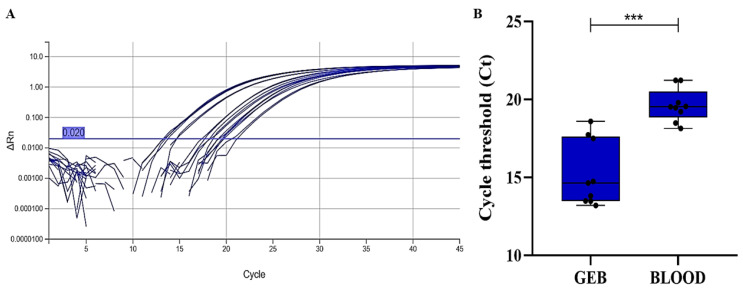
(**A**) Amplification curves for the endogenous control U6 in GEB and whole blood samples. (**B**) Box plot comparing Ct variations of U6 between GEB and whole blood. In (**A**), the blue horizontal line means the qPCR threshold. In (**B**), the circles mean the individual Ct value for each sample. One-way ANOVA *** *p* < 0.001.

Similarly, an endogenous internal control was evaluated, targeting a small endogenous human RNA (U6) (Figure 3A). In this case, significant differences were also observed between GEB (mean Ct 15.246 ± 2.105) and whole blood (mean Ct 19.630 ± 1.052), similar to what was observed with the exogenous internal controls (Figure 3B).

### 3.2. Analysis of mRNA Integrity

Following the evaluation of exogenous and endogenous internal controls for miRNAs, the integrity of messenger RNA was also investigated. Supplementary Figure S1A,B show significant differences in the detection of the human RNase P gene transcript between the two groups analyzed over 120 days of storage. GEB samples (Supplementary Figure S1A) exhibited lower variability in Ct values compared to whole blood samples (Supplementary Figure S1B).

In addition, RNA integrity was assessed by capillary electrophoresis using the TapeStation 4150 system and the High Sensitivity RNA ScreenTape Assay kit, on preservative-free blood and GEB samples stored for 120 days, including a positive control of cardiac tissue processed in parallel. As shown in Supplementary Figure S2, the positive control performed adequately, with an RNA Integrity Number (RIN) value of 8.1 and the presence of the 18S and 28S peaks. These peaks were not detected in the blood samples, with or without guanidine-EDTA, due to the low RNA concentration in blood samples.

Figure S2A shows the gel images corresponding to the electropherograms shown in Figure S2B. While the positive control exhibited well-defined ribosomal RNA bands and no smear, the whole blood and GEB samples showed low RIN values. Nevertheless, the GEB sample exhibited greater relative integrity (RIN = 4.1) compared to the blood sample without a preservative (RIN = 1.7). Furthermore, in the GEB sample, a band consistent with the 18S subunit and lower background intensity were observed, whereas in the samples without preservative, no ribosomal bands were observed, and a more intense background was present.

**Figure 4:**
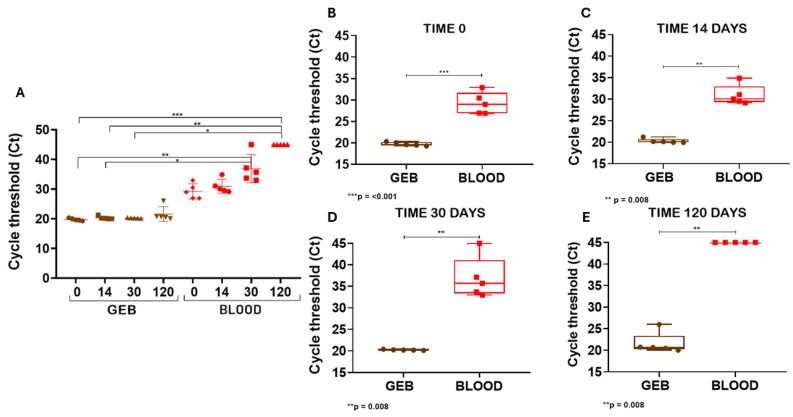
Analysis of mRNA stability in GEB and whole blood, both stored at 4 °C for up to 120 days (Kruskal–Wallis test with Dunn’s post hoc test). (**A**) Scatter plot comparing Ct variations between GEB and whole blood across all time points. (**B**) Box plot comparing Ct variations between GEB and whole blood at day 0 (*p* < 0.001, Mann–Whitney Rank Sum test). (**C**) Box plot comparing Ct variations between GEB and whole blood at day 14 (*p* = 0.008, Student’s *t*-test). (**D**) Boxplot comparing Ct variations between GEB and whole blood at day 30 (*p* = 0.008, Student’s *t*-test). (**E**) Box plot comparing Ct variations between GEB and whole blood at day 120 (*p* = 0.008, Student’s *t*-test). The different symbols mean the individual Ct value for each sample. * *p* < 0.05, ** *p* < 0.01, *** *p* < 0.001.

Complementing these findings, Figure 4A presents the Ct values corresponding to the human RNase P gene transcript over the different storage periods (0, 14, 30, and 120 days post-collection). Statistical analysis was performed using one-way ANOVA followed by multiple comparison tests, revealing significant differences both between the GEB and whole blood groups at each time point and across the storage duration. For the GEB group, no statistically significant differences were observed among the different time points (0, 14, 30, and 120 days), indicating stability of Ct values (~20) over time. In contrast, in the whole blood group, a progressive and significant increase in Ct values was observed starting from day 14 (*p* < 0.05 compared to day 0), with even less efficient amplification at days 30 and 120 (*p* < 0.01 and *p* < 0.001, respectively).

In GEB samples, mean Ct values ranged from 19.75 to 21.58, whereas in whole blood a wider range was observed, with values between 29.25 and 45.0. Figure 4B–E show that, at all evaluated intervals separately, comparisons between the groups revealed statistically significant differences, as indicated by Student’s *t*-test or Mann–Whitney Rank Sum test. It is noteworthy that samples without detectable amplification were assigned a Ct value of 45, corresponding to the maximum number of RT-qPCR cycles. In this context, one whole blood sample in Figure 4D and five whole blood samples in Figure 4E showed no amplification and were treated accordingly. These results demonstrate that, after 120 days of storage, the addition of guanidine-EDTA preserved mRNA integrity in the samples, in contrast to the progressive degradation observed in the absence of the stabilizing agent.

### 3.3. Validation with Samples of Patients with Chagas Disease

**Figure 5:**
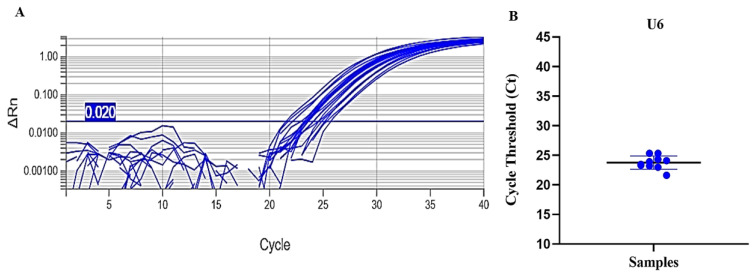
(**A**) Amplification curves of the small RNA U6 in GEB samples stored at 4 °C for 18 years. The blue horizontal line means the qPCR threshold. (**B**) Scatter plot illustrating the variation in Ct values for the U6 target among GEB samples. The blue circles mean the individual Ct value for each sample. The horizontal line means the median for the Ct values.

Finally, the study was validated by analyzing 10 GEB samples obtained from patients diagnosed with CD, stored at 4 °C for a period of 18 years. In this evaluation, endogenous (U6) and exogenous (cel-miR-39 and cel-miR-54) controls were used, in addition to human RNase P as an endogenous marker for mRNA. Figure 5 shows that the mean Ct values for U6 were higher compared to analyses with negative GEB samples (Figure 2); however, the difference did not reach statistical significance, with a minimum value of 21.63, a maximum of 25.33, and a mean ± standard deviation of 23.81 ± 1.07.

**Figure 6:**
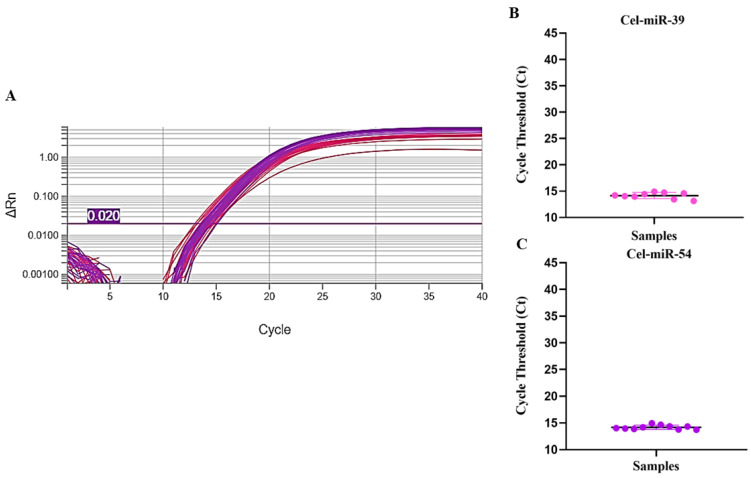
(**A**) Amplification curves of the exogenous miRNAs cel-miR-39 (shown in pink) and cel-miR-54 (shown in purple) in GEB samples stored at 4 °C for 18 years. The pink horizontal line means the qPCR threshold. (**B**) Scatter plot illustrating the variation in Ct values for cel-miR-39 among GEB samples from the 10 patients evaluated in this study. The pink circles mean the individual Ct value for each sample. The horizontal line means the median for the Ct values. (**C**) Scatter plot showing the variation in Ct values for cel-miR-54 among GEB samples from the 10 patients evaluated in this study. The purple circles mean the individual Ct value for each sample. The horizontal line means the median for the Ct values.

For the exogenous internal controls, descriptive analysis showed a consistent distribution of Ct values, as illustrated in Figure 6. For cel-miR-39, the median was 14.32, with 1st and 3rd quartiles of 14.00 and 14.69, respectively, resulting in an interquartile range (IQR) of 0.69. The upper detection limit, defined as 75% + 1.5 × IQR, was estimated at 15.72. For cel-miR-54, the median was 14.09, with quartiles of 13.88 (25%) and 14.33 (75%), corresponding to an IQR of 0.45 and an upper limit of 15.00. These results indicate low variability and no relevant outliers for both controls.

**Figure 7:**
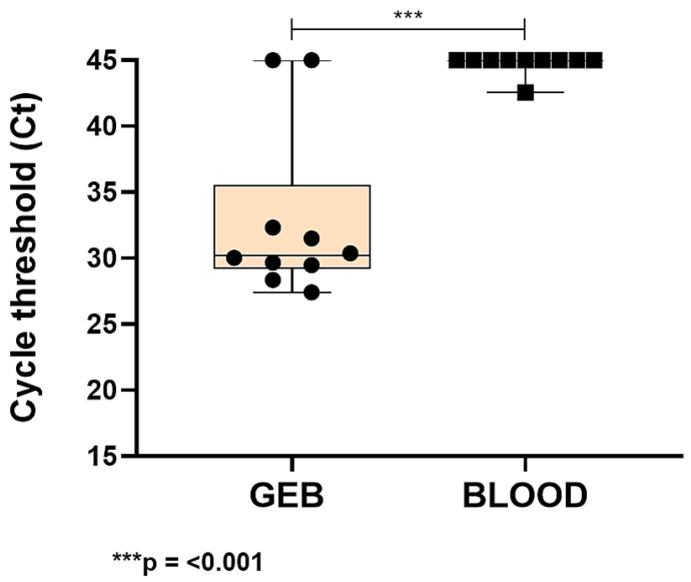
Box plot comparing Ct values obtained for the constitutive RNase P gene after mRNA extraction from whole blood, with (GEB) and without the addition of guanidine-EDTA (Blood). The circles and squares mean the individual Ct value for each sample. *** *p ≤* 0.001, Student’s *t*-test.

Finally, we evaluated the endogenous RNase P target in 10 GEB samples obtained from patients with Chagas disease (CD), stored at 4 °C for 18 years, and compared them with 10 blood samples from CD-negative individuals that were not mixed with guanidine-EDTA and stored at 4 °C for 120 days, in order to investigate mRNA detection. Comparative analysis of the mean Ct values revealed a statistically significant difference in mRNA detection between samples preserved in GEB and those stored without a stabilizing agent (*p* < 0.001; Figure 7). These findings demonstrate that the absence of guanidine-EDTA compromised mRNA detection in blood samples, highlighting the importance of this stabilizing agent for maintaining mRNA integrity and stability during storage.

## 4. Discussion

The use of guanidine-EDTA for blood sample preservation represents a key approach to safeguarding nucleic acid integrity, playing a critical role in enabling reliable molecular diagnosis of Chagas disease and effective monitoring of patient response to therapy. Guanidine-HCl, a chaotropic salt, plays a fundamental role in protein denaturation and nuclease inhibition, resulting in the effective preservation of DNA and RNA in biological samples [6].

Moreover, the ease of sample transport without the need for refrigeration is especially advantageous in remote areas, where access to specialized laboratories may be limited. Another relevant aspect is the sample volume, which becomes a critical variable in the detection of *T. cruzi*, whose distribution in blood is not homogeneous. This issue can be efficiently addressed by adding guanidine-EDTA to blood samples in a 1:1 ratio. Research indicates that larger volumes of blood, ranging from 2 to 5 mL, significantly increase the likelihood of parasite detection, particularly in patients with chronic infection [25,26].

Previous studies have demonstrated the effectiveness of guanidine in DNA extraction. For example, Ávila et al. (1991) [6] documented the amplification of *T. cruzi* minicircle DNA isolated from whole blood lysates, showing that the use of guanidine-EDTA contributed to optimized performance in parasite detection. They confirmed that *T. cruzi* DNA can be efficiently extracted from samples stored in guanidine-HCl/EDTA solution, maintaining its integrity even after incubation at 37 °C for up to one month. However, recent studies have demonstrated that *T. cruzi* RNA undergoes rapid degradation, whereas DNA detection remains comparatively stable, highlighting the critical importance of appropriate preservation strategies for both types of nucleic acids [27].

In this context, our findings show that guanidine-EDTA effectively preserves the integrity of messenger RNA and microRNAs, even when blood samples stored in guanidine-EDTA buffer are maintained at 4 °C for prolonged periods. This preservation is of utmost importance, as RNA molecular analysis poses additional challenges compared to DNA analysis, due to its labile nature and the rapid degradation caused by RNases present in biological samples. Therefore, the preservation of total RNA under appropriate conditions, including the addition of RNase inhibitors, becomes essential to ensure reliable results in the molecular diagnosis of CD, particularly with respect to differentiating viable from non-viable parasites in the samples [28,29].

Additionally, a study conducted by Farani et al. (2023) [30] analyzed the miRNA transcriptome in the cardiac tissue of mice chronically infected with *T. cruzi*. In this study, 641 miRNA targets were investigated in mice treated and untreated with BZN during the chronic phase of experimental disease. The results revealed 20 miRNAs whose expression levels were significantly increased or decreased in the untreated infected group, returning to levels considered normal after BZN treatment. These findings suggest that host miRNAs may serve as biomarkers of therapeutic response in patients with chronic CD. Thus, the identification of biomarkers to monitor therapeutic efficacy in CD has become an area of intense investigation. In the present study, we also evaluated the ability of guanidine-EDTA to preserve these miRNA molecules in blood samples.

In the initial analysis, blood samples negative for *T. cruzi* were tested with and without the addition of guanidine-EDTA. Under both conditions, the same known volume and concentration of exogenous miRNA controls were added to assess the performance of these controls under different preservation conditions. The results showed that blood samples preserved in GEB exhibited higher sensitivity compared to pure blood samples. It was observed that the Ct values for the exogenous internal control targets were significantly lower in GEB samples than in pure blood samples, indicating that guanidine not only enhances amplification efficiency but is also crucial for the accurate detection of microRNAs, especially in patient blood samples.

The use of exogenous controls in miRNA expression analyses is widely recommended in scientific literature, as it allows correction of variations introduced throughout the experimental steps, such as extraction efficiency, material loss during processing, and differences in reverse transcription or amplification yield. By being added at known concentrations before extraction, these controls act as internal references independent of the patient’s biological material, ensuring greater reliability in data normalization and enabling comparison across different samples and experimental conditions [31,32]. Furthermore, in biological fluids, where the amount of RNA is limited and interindividual variability is high, exogenous controls become essential to distinguish true alterations in miRNA expression from technical artifacts or processing failures.

We also evaluated the same samples for the small RNA U6, widely recognized as an endogenous internal control in miRNA expression analyses. U6 is a small nuclear RNA (snRNA) involved in pre-mRNA splicing and, due to its relatively abundant expression, is often used as a reference in many studies [31,33]. In the present study, U6 showed minor variations among amplifications within the same group but overall maintained lower Ct values in blood samples mixed with guanidine-EDTA compared to pure blood samples.

The inclusion of U6 as an endogenous control was fundamental, as it allows the assessment of whether the guanidine-EDTA preservation protocol impacts not only the exogenous material but also the host’s endogenous cellular RNA. Unlike exogenous controls, which monitor technical efficiency, U6 reflects the stability of RNA naturally present in blood cells. Studies such as Xiang et al. (2014) [34] have shown that U6 can vary considerably in circulating samples, especially under freeze–thaw conditions, suggesting that it is not always reliable as a universal normalizer in plasma or serum. It is important to note, however, that U6 expression can be influenced by biological factors such as age, health status, and associated diseases, as well as by technical factors such as sample collection, processing, and storage, which may affect the Ct values obtained. The literature suggests that, in many cases, the use of more than one endogenous control, or prior validation of controls for the specific batch of samples under study, contributes to greater reliability of the data [35,36,37,38].

Moving on to messenger RNA analysis, we performed a systematic comparison of the preservation of GEB and pure blood samples over time, considering a period of up to 120 days (4 months) post-collection. The choice of RNase P as a target was strategic, since it is a constitutively expressed gene widely used as an endogenous control in molecular assays, precisely because it reflects the integrity of human messenger RNA present in the sample [39,40]. We observed that the use of guanidine-EDTA was effective in preserving mRNAs in blood samples, in contrast to the pronounced degradation observed in the absence of the preservative agent. While GEB samples exhibited consistent Ct values for RNase P around 20 throughout the 120-day period, pure blood samples showed a progressive increase in Ct values, ultimately resulting in the inability to detect the transcript after this interval. Similar results have been reported in studies evaluating mRNA stability in whole blood, which describe rapid degradation in the absence of RNase inhibitors [41], in contrast with the preservation promoted by the use of chaotropic agents such as guanidine [42]. Accordingly, the use of RNase P as an endogenous control serves both to confirm RNA integrity and to further support the suitability of guanidine-EDTA for long-term preservation of biological samples. However, the observed differences in Ct values between GEB and blood samples may not be exclusively attributed to RNA preservation, but also to additional factors inherent to the sample matrix, including the presence of PCR inhibitors in whole blood, differences in extraction efficiency, and the enhanced cell lysis and nucleic acid release promoted by guanidine-based buffers; these combined effects should be considered when interpreting the magnitude of Ct variation.

Additionally, the analysis of RNA integrity by capillary electrophoresis in these samples provides a complementary perspective to the findings obtained by RT-qPCR. The absence of 18S and 28S peaks in blood samples, regardless of the presence of guanidine-EDTA, is likely associated with the low total RNA concentration in this type of sample, which may impact RIN determination. This factor may also explain the RIN values below the recommended threshold, even in preserved samples. Nevertheless, GEB samples showed relatively higher integrity, as indicated by higher RIN values, the presence of a band compatible with the 18S subunit, and lower smear intensity. In contrast, samples without preservative agent showed no detectable ribosomal bands and a more pronounced degradation pattern. Taken together, these findings suggest that the presence of guanidine-EDTA may contribute to RNA preservation during storage, although this interpretation should be considered in light of the inherent limitations of the sample type and the methodology used.

Finally, we validated samples from patients with Chagas disease that had been stored in guanidine-EDTA for 18 years and previously subjected to boiling. This step, commonly employed in older protocols for conventional PCR targeting kDNA, was intended to promote minicircle network deconcatenation and enhance diagnostic sensitivity [8]. Notably, even after boiling and prolonged storage, mRNA as well as endogenous and exogenous miRNAs were successfully detected, showing consistent Ct values and low variability, which indicates adequate molecular preservation.

In contrast, samples that were not subjected to boiling and were stored without guanidine-EDTA, even for a substantially shorter period (120 days), showed marked impairment in mRNA detection, as evidenced by reduced amplification of the constitutive human RNase P gene. These findings highlight the critical role of preservation conditions in maintaining RNA integrity.

Importantly, the use of boiling has been discontinued in current protocols. With the adoption of genomic targets such as *T. cruzi* nuclear satellite DNA, this step is no longer required, in accordance with international consensus recommendations for molecular diagnosis by qPCR [10]. Consistently, our experience indicates that boiling guanidine-EDTA lysates is unnecessary when targeting satDNA.

Despite these methodological differences, both boiled and non-boiled samples were included in the present study and yielded consistent and comparable results. This suggests that the boiling step does not impair miRNA detection and supports the use of archived samples for biomarker studies. In addition, these results reinforce the role of guanidine as a chaotropic agent capable of inactivating RNases and preserving transcript integrity. The statistically significant difference observed between preserved and non-preserved samples (*p* < 0.001; Figure 7) further underscores the importance of stabilization agents for RNA-based analyses under prolonged storage conditions.

Although the stability of *T. cruzi* DNA in GEB has been previously reported [6,25,26], evidence regarding the long-term preservation of RNA and miRNAs remains limited. In this context, our findings expand the applicability of guanidine-EDTA, supporting its use not only for molecular diagnosis of Chagas disease but also for therapeutic monitoring strategies based on RNA biomarkers. This opens new perspectives for the use of previously stored GEB samples in gene expression and miRNA studies, both in Chagas disease and potentially in other conditions. However, a limitation of the present study is the absence of direct comparisons with other established RNA stabilization systems. Future studies addressing the comparative performance of guanidine-EDTA and these other commercial reagents under different experimental and clinical conditions will be important to further define the relative advantages, limitations, and potential applications of this preservation approach.

## 5. Conclusions

The present study consistently demonstrates that the addition of guanidine-EDTA to human blood constitutes a highly effective strategy for the preservation of nucleic acids, including mRNA and miRNAs. Samples preserved in this solution exhibited greater analytical sensitivity, lower variability in Ct values, and sustained transcript integrity over time, in contrast to the progressive degradation observed in untreated blood samples.

Furthermore, the stability of endogenous (U6) and exogenous (cel-miR-39 and cel-miR-54) miRNA controls and RNAse P transcript confirms that the guanidine-EDTA buffer preserves not only experimentally added synthetic molecules but also RNAs naturally present in biological samples.

For the first time, we validated the effectiveness of this approach in samples from patients with CD stored at 4 °C for 18 years, in which mRNA and microRNAs were successfully detected with consistency and low variability. These findings underscore the value of guanidine-EDTA as a strategy for long-term sample preservation, supporting retrospective molecular investigations and biomarker-based approaches for therapeutic monitoring. Taken together, our results demonstrate that preserving blood in guanidine-EDTA maintains nucleic acid integrity over extended periods while broadening its utility for high-sensitivity molecular diagnostics and the development of novel tools for monitoring Chagas disease. However, for prolonged transport at room temperature, additional studies are still required to validate this condition.

## Data Availability

The original contributions presented in this study are included in the article/Supplementary Materials. Further inquiries can be directed to the corresponding authors.

## Supplementary Materials

The following supporting information can be downloaded at: https://www.mdpi.com/article/10.3390/pathogens15040424/s1, Figure S1: Detection of the human RNase P transcript in blood samples preserved or not in guanidine-EDTA at 4 °C for 120 days. Figure S2: RNA integrity analysis carried out with Agilent 4500 TapeStation system.
