# Supplementary material for: Validation of Guanidine-EDTA as a Preservative Agent for the Analysis of miRNAs and mRNAs in Blood Samples of Chagas Disease Patients
Source: Pathogens. 2026 Apr 14;15(4):424. doi: 10.3390/pathogens15040424 (PMC13118385; doi:10.3390/pathogens15040424)
Supplement: Supplementary file 1 [file pathogens-15-00424-s001.zip › pathogens-4204543-supplementary.pdf]

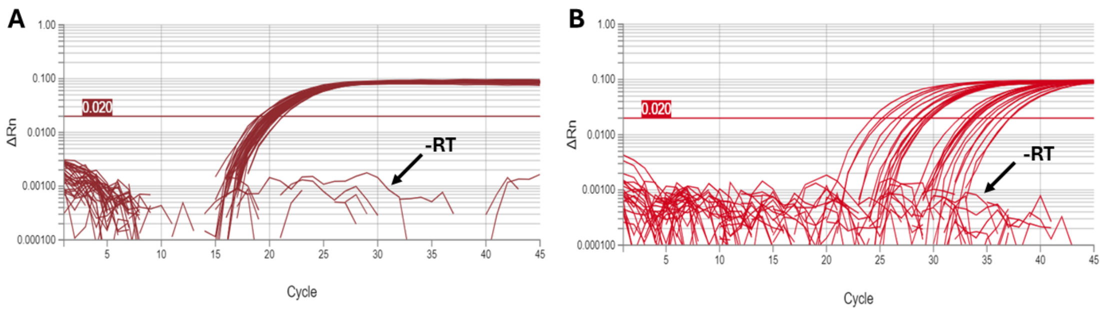

Supplemental Figure 1. Detection of the human RNase P transcript in blood samples preserved or not in guanidine-EDTA at 4 °C for 120 days. A) Amplification curves of RNase P in GEB samples. B) Amplification curves of RNase P in whole blood samples without guanidine-EDTA. –RT reactions were included as negative controls.

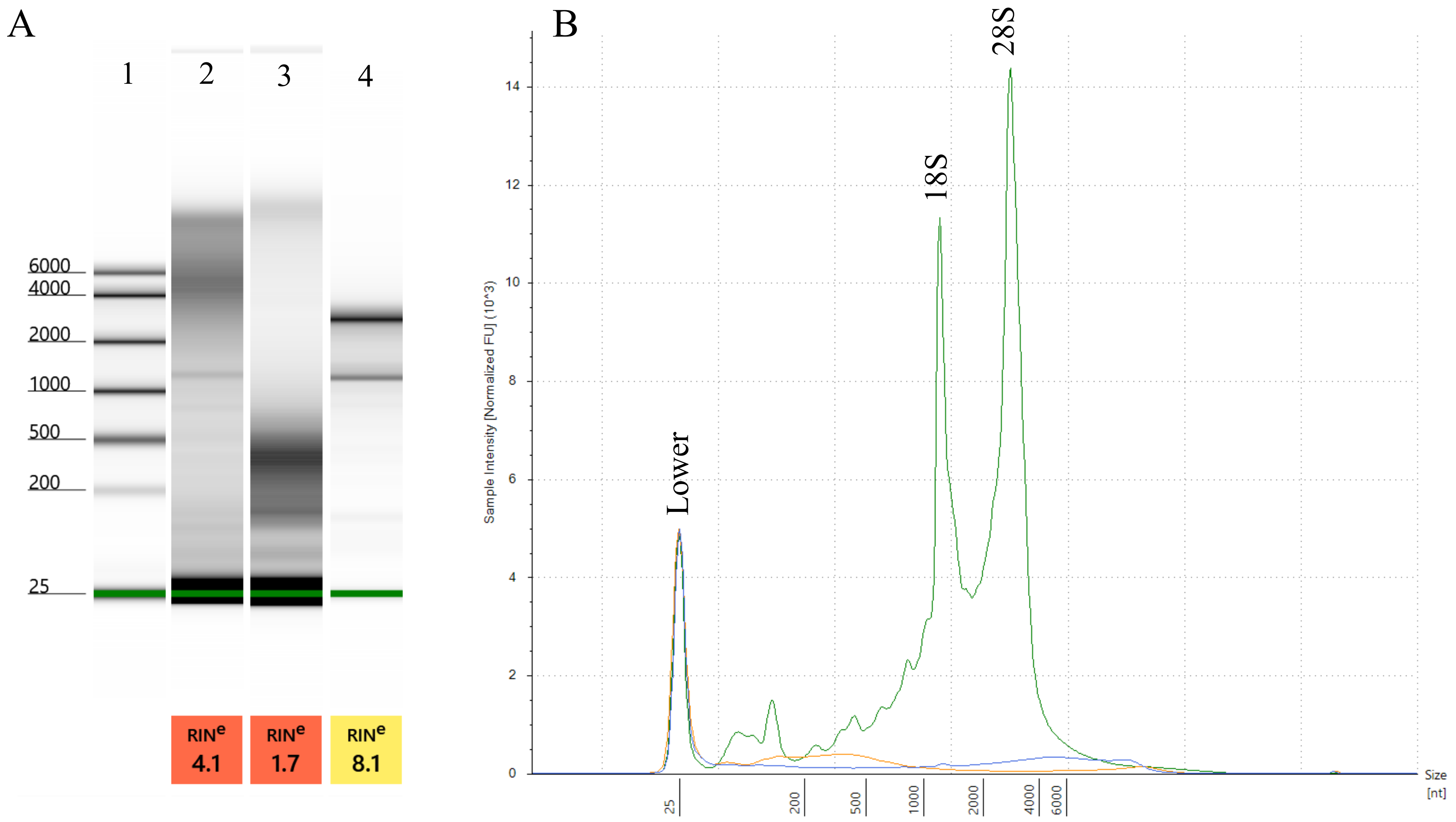

Figure S2. RNA integrity analysis carried out with Agilent 4500 TapeStation system. A) The gel image shows the separation profile of each sample along with the RINe (below). Lanes: 1- Ladder. 2- GEB 120 Days. 3- Blood 120 Days. 4- Positive control (mice heart tissue). B) Electropherogram overlays of GEB 120 Days (blue line), Blood 120 Days (orange line) and positive control (mice heart tissue, green line) RNA samples. To the positive control, the electropherogram shows ribosomal RNA peaks of 28S and 18S, along with the lower marker.
